# Oxidation and Sulfidation Resistance of Hot-Pressed AlCrMoTaTi and AlCrNbTaTi Alloys at High Temperatures

**DOI:** 10.3390/ma19112364

**Published:** 2026-06-02

**Authors:** Grzegorz Smoła, Paweł Gradoń, Richard Gaweł, Grzegorz Moskal, Zbigniew Grzesik

**Affiliations:** 1 Department of Physical Chemistry and Modelling, Faculty of Materials Science and Ceramics, AGH University of Krakow, al. A. Mickiewicza 30, 30-059 Krakow, Poland; smola@agh.edu.pl (G.S.); ragaw@agh.edu.pl (R.G.); 2 Materials Innovation Laboratory, Silesian University of Technology, Krasińskiego Str. 8, 40-019 Katowice, Poland; pawel.gradon@polsl.pl (P.G.); grzegorz.moskal@polsl.pl (G.M.)

**Keywords:** refractory metals, high-entropy alloys, hot pressing, oxidation, sulfidation

## Abstract

A metallic material resistant to the attack of both oxygen and sulfur at high temperatures has been desired for years. Unfortunately, commonly used commercial oxidation-resistant materials degrade rapidly in the presence of sulfur. Conversely, sulfidation-resistant metals oxidize at a rate that eliminates their practical use in oxygen-containing atmospheres. However, the latest studies in this area strongly suggest that some refractory high-entropy alloys produced by arc melting can be resistant to oxidation and sulfidation to a degree comparable to that of several traditional materials currently used in individual gas environments. Nevertheless, melting refractory metals require high temperatures and expensive equipment. To eliminate these difficulties, this work used hot pressing to fabricate the AlCrMoTaTi and AlCrNbTaTi refractory multi-component alloys, the resistance of which was tested thermogravimetrically in atmospheres containing oxygen and sulfur vapors at temperatures ranging from 800 to 1000 °C. The achieved results clearly indicate that although these alloys are not homogeneous, they exhibit high resistance. In general, both alloys react in accordance with the parabolic law, with the AlCrMoTaTi alloy exhibiting greater resistance than the AlCrNbTaTi counterpart. Consequently, these alloys can potentially be used not only as solid materials but also in coatings produced, for example, by plasma spraying.

## 1. Introduction

The diverse chemical composition of high-temperature aggressive industrial atmospheres is the reason a single metallic material is incapable of operating under all conditions [1,2,3]. This is because oxidation-resistant alloys (i.e., chromia and alumina formers) degrade rapidly in atmospheres containing, for example, sulfur, while metals with high melting points, which are resistant to sulfidation, corrode rapidly in oxygen atmospheres [4,5,6,7]. Consequently, the prevailing opinion has been that it is impossible to create a material that is resistant to the simultaneous attack of oxygen and sulfur. Therefore, precise selection of alloy compositions for operation in precisely defined corrosive atmospheres is necessary.

It is worth emphasizing that, in the past, great efforts have been made to change this situation. Among the most promising was the development of binary amorphous alloys consisting of aluminum with selected high-melting metals [7,8,9,10,11]. Unfortunately, although these alloys exhibited very good sulfidation resistance, exceeding that of molybdenum and niobium, their oxidation resistance remained limited. The best amorphous alloys among the studied materials, i.e., Al-(34–46)Mo alloys, demonstrated fully satisfactory oxidation resistance only up to a temperature of approximately 850 °C. This was due to the formation of a protective Al_2_O_3_ scale layer [7]. The formation of large amounts of volatile molybdenum oxides above this temperature directly caused the alloys to degrade rapidly [8]. An attempt to improve the oxidation resistance of amorphous alloys by adding 6 at.% of silicon proved effective only up to approximately 950 °C [7]. Increasing the silicon content, on the other hand, while improving oxidation resistance, dramatically increased the sulfidation rate. Furthermore, these alloys became extremely brittle. All these difficulties prevented binary amorphous aluminum alloys with high-melting metals from being a viable solution to this problem.

However, recent research indicates that it is possible to select the composition of heat-resistant high-entropy alloys (AlCrMoTaTi) to ensure a relatively low oxidation rate even up to 1400 °C [12], while also ensuring slow degradation in a sulfur vapor environment [13]. It appears, therefore, that high-entropy heat-resistant alloys built of aluminum, chromium, and high-melting metals represent a breakthrough in the search for a material resistant to complex gas atmospheres containing oxygen and sulfur at high temperatures. Up to now, these alloys have been obtained by repeated melting in an electric arc furnace. Due to the high melting temperatures of molybdenum and tantalum, this alloying method is cumbersome and, in many cases, limits the production of components with specific shapes. Consequently, in this study, it was decided to produce such materials by hot pressing of the appropriate metal powders.

Thus, the aim of this work was to determine the oxidation and sulfidation behavior of AlCrMoTaTi and AlCrNbTaTi alloys obtained in such a manner in the temperature range 800 to 1000 °C, using the microthermogravimetric technique. Both alloys were oxidized in air, as well as sulfidized in a He-S_2_ gas mixture at the sulfur vapor pressure of 1000 Pa.

## 2. Materials and Methods

The AlCrMoTaTi and AlCrNbTaTi alloys used in the study were equiatomic alloys with a 20% atm content of each component. Pure metal powders (Chemat, Konin, Poland) with a manufacturer-declared purity of 99.98% were used in the alloy production process. All powders were characterized by a nearly spherical granule shape and an average granule diameter of approximately 45 µm. Prior to consolidation, the powders were mechanically mixed in a turbulent mixer at 60 rpm for 60 min. This was done to homogenize the feedstock composition throughout the powder mixture and to break up any agglomerated particles.

**Figure 1:**
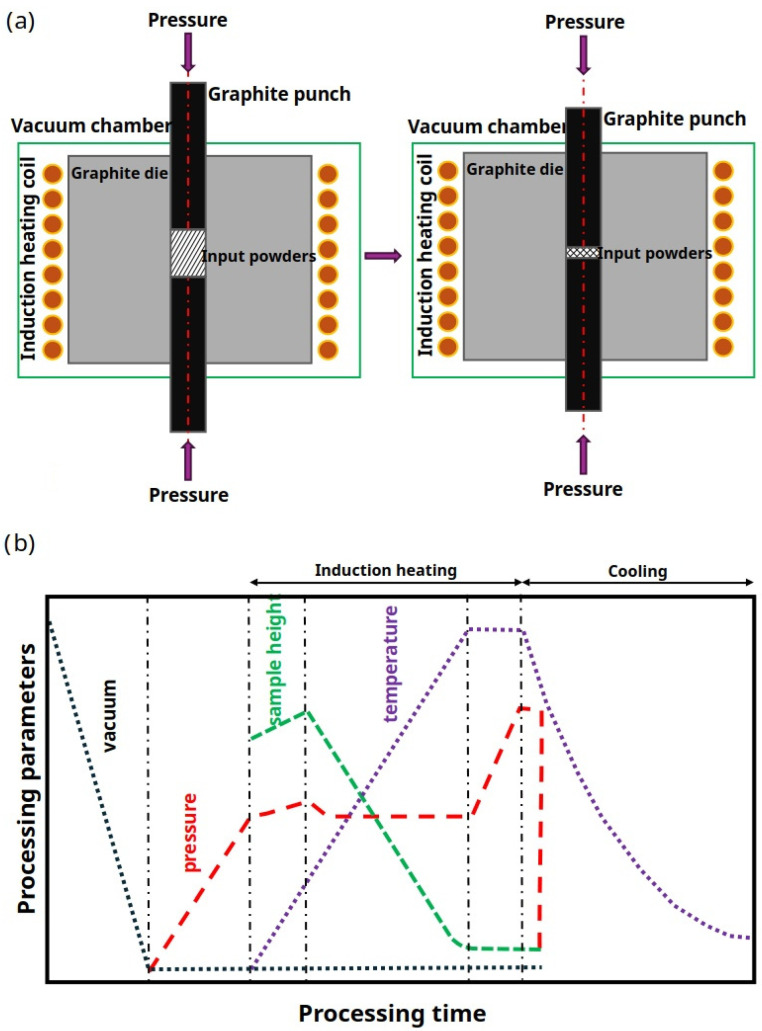
Schematic representation of the (**a**) consolidation and sintering process and (**b**) their basic parameters.

To consolidate the powders, a hot-vacuum pressing method was used, followed by sintering in a Degussa VSPi 15/20 press (Degussa AG, Frankfurt, Germany). The process was carried out under vacuum conditions of 2.67 × 10^−6^ MPa at a temperature of 1200 °C and a pressure equal to 15 MPa. This is the highest pressure the equipment is capable of providing, allowing for the lowest possible porosity in the received materials. The sintering time was 1 h. Graphite dies were used to produce cylinder-shaped samples with a 25 mm diameter and a thickness that depended on the amount of powder introduced. In this case, the final cylinder thickness was 15 mm. The diagram of the consolidation and sintering process, as well as its basic parameters (i.e., temperature, vacuum, time, and pressure), is shown in Figure 1.

The relative density of the sinters was determined using the hydrostatic method. Its values were 99.78% and 99.82% for the AlCrMoTaTi and AlCrNbTaTi alloys, respectively, corresponding to porosities of 0.22% and 0.18%.

From the prepared material, 1 mm thick disc-shaped slices were cut using the electrospark method (perpendicular to the sinter axis) for the corrosion tests. Each slice was cut into four identically sized segments, with 2 mm diameter holes drilled in the corners to allow the samples to be hung in the testing apparatus on a specially prepared quartz suspension. The received samples were then subjected to grinding using sandpapers (Struers, Ballerup, Denmark) ranging from #180 to #1000 gradation and subsequently polished with the help of diamond pastes, the particles sizes of which were 9, 3 and 1 µm, respectively. As a result, samples for studies with surface area ranging between 3 and 3.5 cm^2^, 1.5–2.5 g mass and up to 1 mm in thickness were obtained. Finally, organic impurities were removed from the samples by immersing them in ethanol inside an ultrasonic cleaner for 10 min and then drying them in a stream of warm air.

High temperature corrosion tests in oxidizing, as well as sulfidizing conditions, were performed under isothermal conditions at three temperatures, namely 800, 900 and 1000 °C, carried out for 100 h, each. In both cases, a microthermogravimetric apparatus equipped with an electronic measuring head with 10^−7^ g sensitivity provided by CI Precision (Salisbury, UK), was utilized. Using this method, kinetic curves for the studied alloys were obtained at the above-mentioned temperatures by recording, in situ, sample mass changes as a function of time. Details on the system used for oxidation tests, along with a schematic illustration, can be found in [14], while details on the thermogravimetric apparatus specifically designed for sulfidation of metallic materials are included in [15]. The use of the latter setup allowed not only accurate measurements of sample mass changes, but also precise control over thermodynamic conditions during the experiments. It is important to note that sulfidation of prepared alloy samples was conducted in an atmosphere consisting of sulfur vapor mixed with high-purity helium (99.9999%, Air Liquide, Kraków, Poland) to ensure a sulfur partial pressure of 10^3^ Pa, while the total reaction atmosphere pressure in the apparatus was 10^5^ Pa. The flow of this atmosphere through the apparatus reaction chamber, where the sample was suspended during the process, was set to 10 mL/min.

The surface morphology and chemical compositions of the samples before oxidation were studied by means of scanning electron microscopy combined with energy-dispersive X-ray spectroscopy (SEM-EDS; Scios 2, ThermoFisher Scientific, Waltham, MA, USA). This was also used to assess the homogeneity of materials obtained by hot pressing, as opposed to the more traditional arc-melting synthesis method. The same SEM-EDS investigations were performed on the samples after the respective high-temperature oxidation and sulfidation processes. Additionally, the phase compositions of the initially obtained alloys and corrosion reaction products were identified by X-ray diffraction (XRD) analysis (X’Pert PRO, PANanlytical, Worcestershire, UK). After that, the samples were mounted in thermosetting resin, cut in half, and subsequently polished with the previously mentioned diamond pastes for cross-sectional SEM-EDS studies.

## 3. Results

### 3.1. Analysis of the Starting Materials

**Figure 2:**
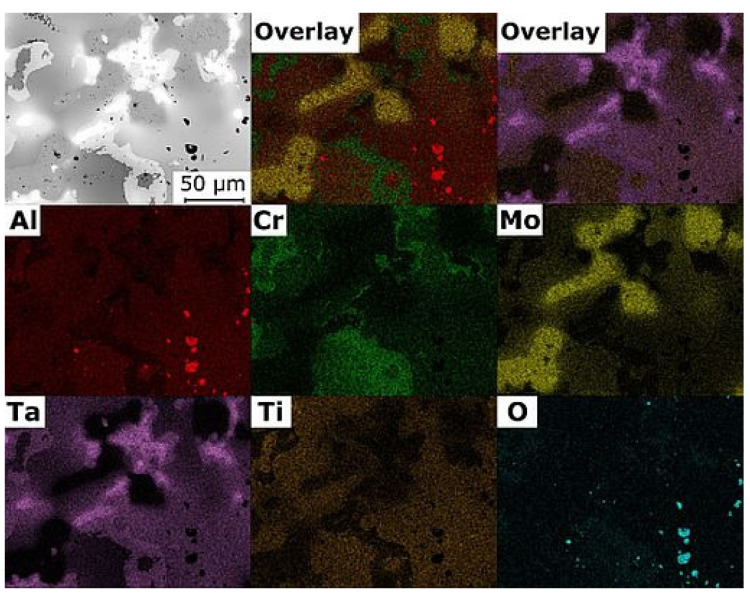
SEM images and EDS maps of hot-pressed AlCrMoTaTi alloy.

**Figure 3:**
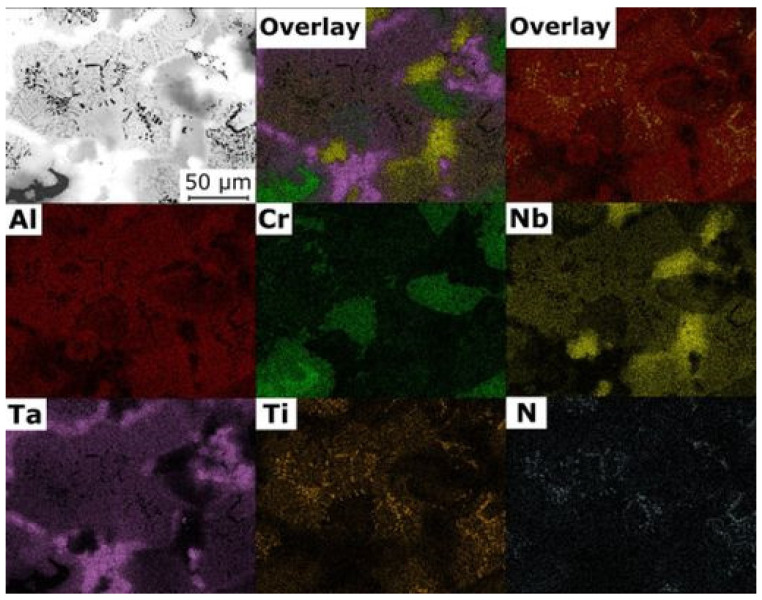
SEM images and EDS maps of hot-pressed AlCrNbTaTi alloy.

Combined SEM-EDS studies revealed that the microstructures of the studied alloys are non-homogeneous, as shown by the element distributions of AlCrMoTaTi and AlCrNb-TaTi in Figure 2 and Figure 3, respectively. In both cases, all the elements appear to be mostly separated, indicating a lack of high-entropy solid-solution phases. Consequently, these materials should be classified as multi-component alloys rather than true HEAs.

**Figure 4:**
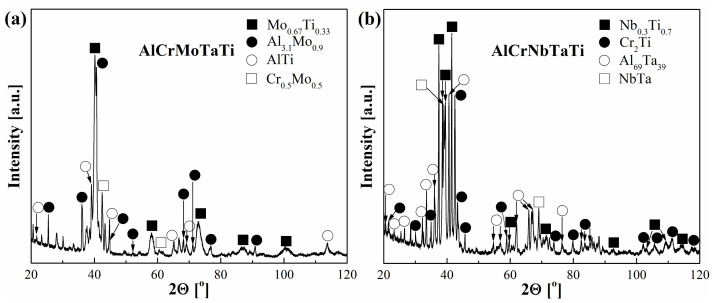
XRD pattern received from the initial hot-pressed (**a**) AlCrMoTaTi and (**b**) AlCrNbTaTi alloy before high-temperature corrosion studies.

The lack of solid solution phases is further confirmed by the XRD patterns obtained from AlCrMoTaTi and AlCrNbTaTi, presented in Figure 4a and Figure 4b, respectively. From these figures it follows that the hot-pressed alloys are primarily composed of two-component intermetallic structures. The PDF numbers of the matching phases are provided in Appendix B, Table A3 and the (*hkl*) indices pertaining to the intermetallic reference structures matching the peaks present in the respective XRD patterns are listed in Table A5, Table A6, Table A7, Table A8, Table A9, Table A10, Table A11 and Table A12.

Analysis of the AlCrMoTaTi alloy indicates a relatively uniform distribution of aluminum across the alloy microstructure. Slightly less uniformity was identified for tantalum and titanium, while chromium and molybdenum exhibit the lowest tendency to homogenize. The overlap between the areas enriched in chromium and those enriched in tantalum is worth noting. This suggests the possible presence of phases, such as the Laves Cr-Ta C15 phase (resulting from high-temperature C14 phase transformation) [16], consistent with the CALPHAD simulation results. Similar observations were also made for areas enriched in titanium, chromium, and molybdenum. This may suggest the presence of Laves Ti-Cr phases with a C36 lattice (also due to high-temperature C14 phase transformation) [17].

The results of microstructural studies on the AlCrNbTaTi alloy also indicate strong chemical composition segregation. As in the case of the molybdenum-containing alloy, aluminum demonstrated the lowest tendency to segregate, followed by tantalum and titanium. Niobium behaved in a manner similar to molybdenum. The main difference observed in the microstructural studies is the matrix area, which contains elongated precipitates strongly enriched in titanium, with similar contents of other alloying elements. The enrichment of these areas in only titanium, with a constant concentration of Mo, Ta, and Nb, indicates the probable presence of a titanium HCP phase modified with these elements, stabilizing this structural variant of titanium, which results from the Ti-Mo, Ti-Ta, and Ti-Nb equilibrium systems [18,19,20,21]. Moreover, much like the AlCrMoTaTi alloy, chromium-rich areas were identified, accompanied by simultaneous increases in tantalum and titanium concentrations, which may indicate the presence of Laves or σ structures.

### 3.2. Oxidation Studies

**Figure 5:**
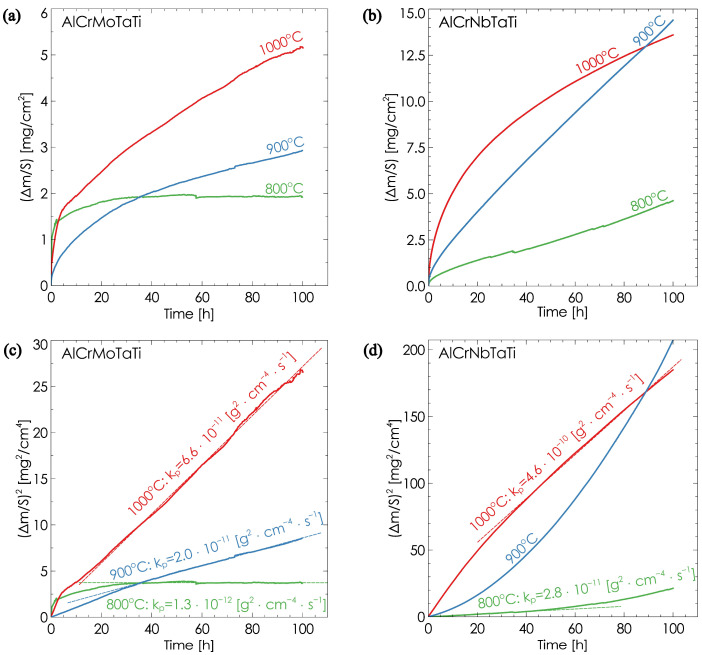
Oxidation kinetic curves of studied alloys, presented in a linear (**a**,**b**) and in a parabolic system of coordinates (**c**,**d**).

The oxidation kinetics curves received from AlCrMoTaTi and AlCrNbTaTi at 800, 900 and 1000 °C are illustrated in Figure 5a and Figure 5b, respectively. These kinetics are also presented in a parabolic coordinate system in Figure 5c,d.

Figure 5 clearly shows that the AlCrMoTaTi alloy oxidizes according to the parabolic rate law over the entire temperature range after a certain initial period. The oxidation of the AlCrNbTaTi alloy, in turn, can be described as parabolic only for the kinetics obtained at 1000 °C. At other temperatures, the oxidation course of this alloy is approximately paralinear, as illustrated in Figure 5d. From Figure 5a,b it also follows that mass gain is significantly higher in the case of the Nb-containing alloy compared to the Mo-rich counterpart. In fact, the total mass gain after 100 h for AlCrMoTaTi at 1000 °C is comparable to that of AlCrNbTaTi at 800 °C.

**Figure 6:**
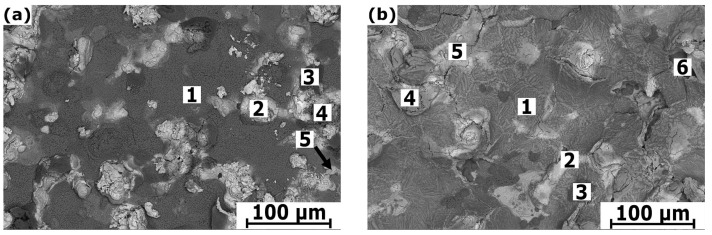
SEM surface images of (**a**) AlCrMoTaTi and (**b**) AlCrNbTaTi after oxidation at 900 °C for 100 h in air. EDS analysis was performed at the indicated points.

The oxide scales grown on both investigated alloys exhibit complex structures. As shown in Figure 6 and Table 1, the SEM-EDS surface analysis of scales obtained at 900 °C indicates that their chemical composition differs significantly among individual scale fragments. This situation is not surprising, considering the heterogeneity of the alloys used for testing. What is somewhat surprising is the presence of relatively large, homogeneous scale fragments in terms of morphological appearance and chemical composition. They are primarily built of Ti-rich oxide. At certain locations, Ta, Al and Cr-rich oxides can also be observed. On the other hand, there are no large areas rich in molybdenum. A similar situation can be observed in the case of scales formed on the AlCrNbTaTi alloy, where mostly Ti, as well as Ta, Nb and Cr-rich oxides are visible. The results of the surface morphology tests performed on the scales obtained during oxidation at other temperatures were analogous to those previously presented.

**Figure 7:**
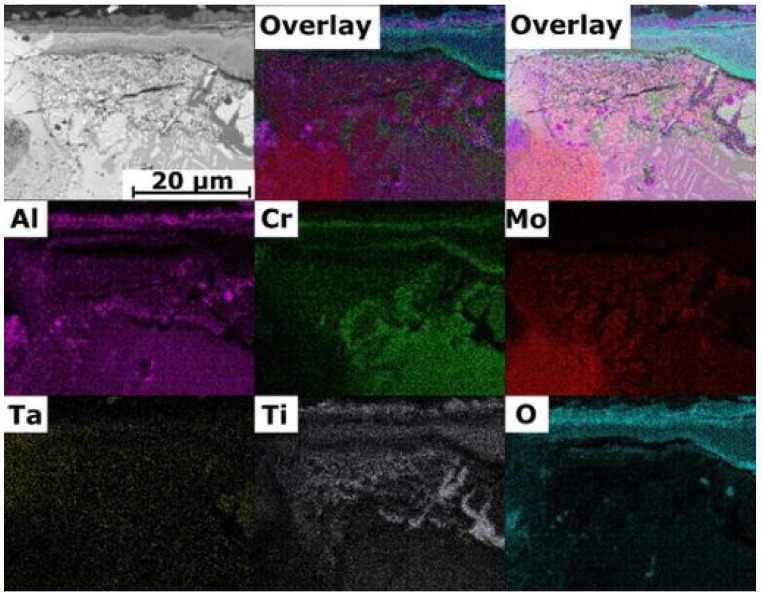
SEM microphotographs and EDS maps of the hot-pressed AlCrMoTaTi alloy after oxidation at 900 °C for 100 h.

**Figure 8:**
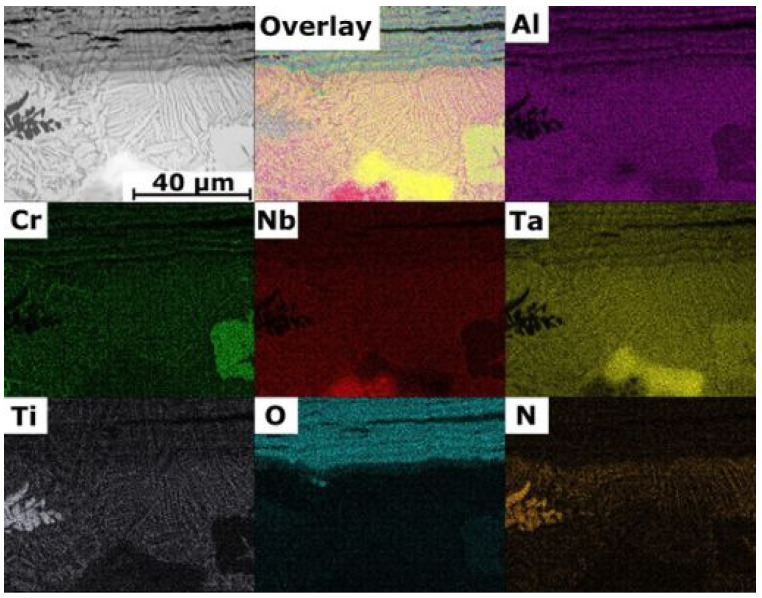
SEM microphotographs and EDS maps of the hot-pressed AlCrNbTaTi alloy after oxidation at 900 °C for 100 h.

It is worth emphasizing that the observations of relatively homogeneous large-scale fragments, determined during the analysis of their surfaces, were also confirmed by the observations of the scale cross-sections shown in Figure 7 and Figure 8. A detailed analysis of the sample cross-sections after oxidation revealed that, in most of the considered cases, the scale is uniform in thickness. Within the reaction product layer, which has a total thickness of 6.31 μm (SD = 0.74, n = 10), three constituent layers can be distinguished. The thicknesses of the outer, middle, and inner layers are 1.50 μm (SD = 0.50, n = 10), 1.19 μm (SD = 0.22, n = 10), and 3.62 μm (SD = 0.39, n = 10), respectively. Nevertheless, there were a few locations where the inner reaction product layer was up to twice as thick, while the thicknesses of the outer and middle layers remained unchanged compared to those observed across most of the surface. Figure 7 presents an example of these investigations illustrating the cross-section of the AlCrMoTaTi alloy after oxidation at 900 °C. It clearly shows the layered structure of the scales, in which individual metals occupy precisely defined positions, despite the heterogeneous nature of the starting material used in the tests. SEM-EDS analysis confirms that the external layer primarily consists of titanium-rich oxide with some Ta visible at the surface. Below that, Al, Cr and Ta-rich layers can be observed. However, the innermost scale layer appears to be a Ti-rich oxide.

Alternatively, the scales grown on the AlCrNbTaTi alloy during oxidation at all temperatures consist of alternating Al, Cr and Ta-rich layers. An example of this is shown in the SEM microphotographs and EDS maps after oxidation at 900 °C (Figure 8).

Significant Nb and Ti content can be observed in the scales as well. Additionally, TiN precipitates are also visible inside the metallic core. In the case of this alloy, the typical lamellar scale structure is clearly visible. The individual lamellae that constitute the scale are difficult to fully distinguish, because there are, at some locations, certain overlaps in the individual metal elements. The most notable overlap is noticed for Ta and Al, indicating a Ti-Al oxide phase. Still, it is not a full overlap, as some Al and Ta content can also be observed separately in the scale, suggesting that Al and Ta-rich oxides are also present.

**Figure 9:**
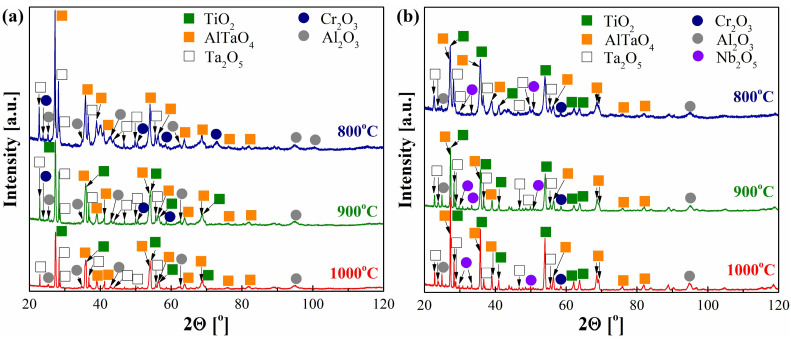
XRD patterns received from hot-pressed (**a**) AlCrMoTaTi and (**b**) AlCrNbTaTi alloy after oxidation at 800, 900 and 1000 °C for 100 h in an air atmosphere.

The XRD patterns obtained for AlCrMoTaTi (Figure 9a) and AlCrNbTaTi (Figure 9b) after oxidation at 800, 900 and 1000 °C confirmed the presence of TiO_2_, Al_2_O_3_, Cr_2_O_3_ and tantalum-containing oxides in the scales, in accordance with the SEM-EDS results. The phase compositions of the scales grown on AlCrMoTaTi and AlCrNbTaTi, respectively, are similar, except for the additional Nb_2_O_5_ identified among the corrosion products of the AlCrNbTaTi alloy. The PDF numbers of the matching phases are provided in Appendix B, Table A4.

It should be noted that the lattice parameters of both Ta-Al spinel oxide and TiO_2_ are very similar, leading to some uncertainty concerning the presence of the spinel phase. However, its existence among the grown oxide layers is consistent with SEM-EDS studies, which indicate Ta and Al-rich formations at the scale surface, below which high Ti content was determined. Therefore, it is possible that selected peaks in the XRD pattern may pertain to both structures. Additional information on the Miller indices corresponding to the individual peaks of the different phases is included in Appendix B, Table A13, Table A14, Table A15, Table A16, Table A17 and Table A18.

### 3.3. Sulfidation

**Figure 10:**
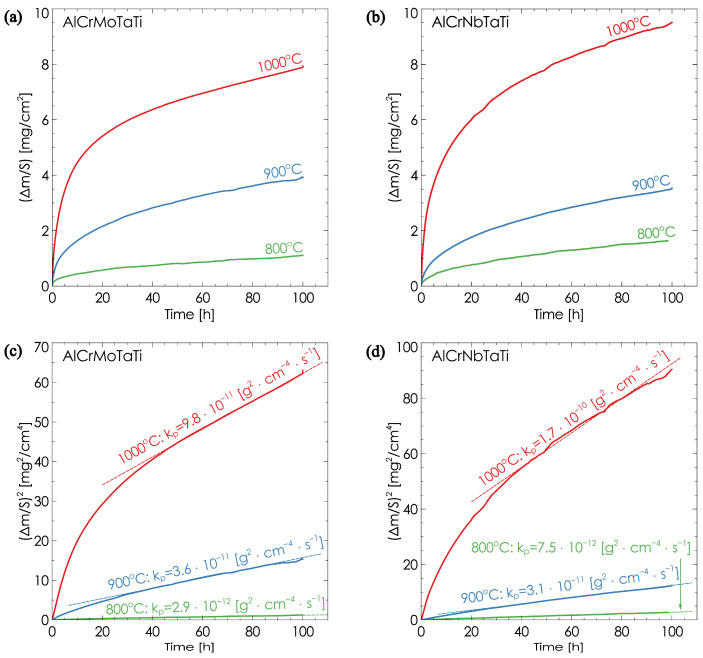
Isothermal sulfidation kinetics of AlCrMoTaTi and AlCrNbTaTi alloys in the temperature range of 800–1000 °C, at a sulfur vapor partial pressure of 1 kPa, plotted in linear (**a**,**b**) and parabolic (**c**,**d**) systems of coordinates, respectively.

The sulfidation process of both studied alloys is presented in Figure 10.

Linear plots (Figure 10a,b) indicate that the sulfidation resistance of both tested alloys is comparable, with a slight advantage for the molybdenum-containing alloy. The latter exhibits marginally lower mass gains per unit area over time in tests conducted at 800 and 1000 °C. At 900 °C, a slightly lower mass gain was recorded for the niobium-containing sample.

**Figure 11:**
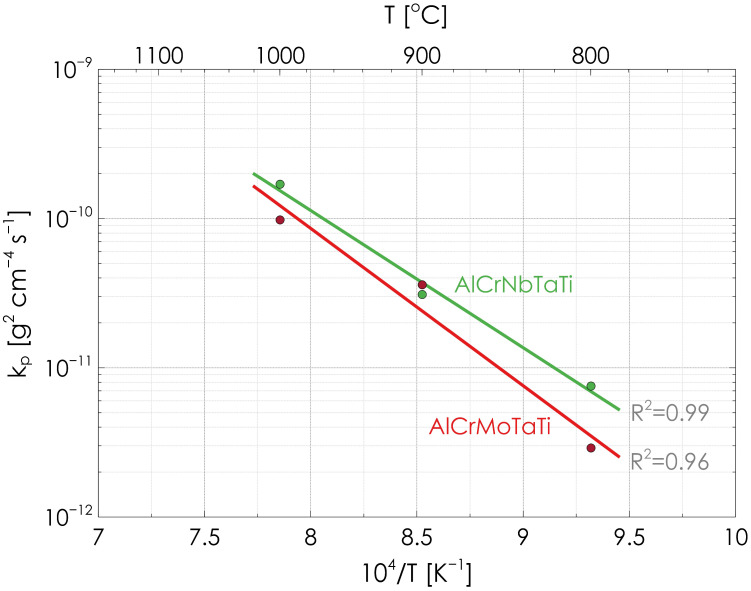
Temperature dependence of the sulfidation rate for AlCrMoTaTi and AlCrNbTaTi alloys.

After an initial transient period, lasting from several to approximately 40 h, the sulfidation of both alloys followed the parabolic rate law (Figure 10c,d). This implies that the growth of the reaction product layers (forming scales) on the surface of the investigated materials is controlled by the diffusion of reactants through these layers. The temperature dependence of the sulfidation rate, plotted using the calculated k_p_ values and shown in Figure 11, clearly demonstrates that this relationship is similar for both alloys.

The apparent activation energy determined from these plots is 202 kJ/mol for the AlCrMoTaTi alloy and 176 kJ/mol for the AlCrNbTaTi alloy, respectively. The similar activation energy values and comparable scale-formation rate characteristics as a function of temperature suggest similar scale formation mechanisms for both alloys. This is further reflected in the morphology of the reaction products covering the surfaces of both materials.

**Figure 12:**
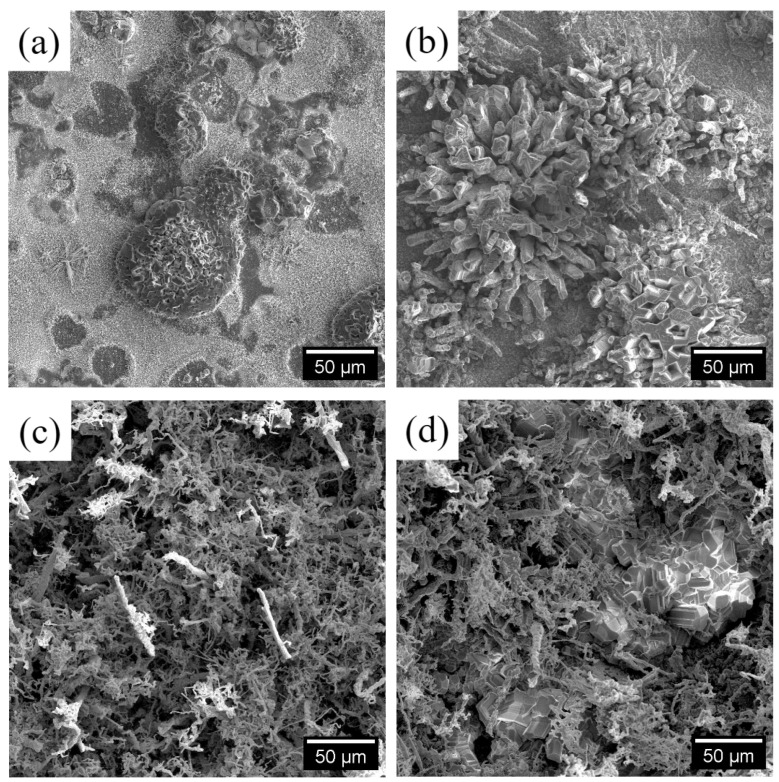
Sulfide scale morphology after 100 h sulfidation of AlCrMoTaTi at (**a**) 800 °C, (**b**) 900 °C, and (**c**) 1000 °C; and (**d**) AlCrNbTaTi at 1000 °C.

Figure 12 illustrates the morphological evolution of the scale forming on the AlCrMoTaTi alloy at temperatures of 800, 900, and 1000 °C.

At 800 °C, the alloy surface is covered with a relatively flat scale, featuring island-like formations (composed of fine, regular crystallites), scattered across the entire surface. At 900 °C, these are replaced by clusters of coarse-grained, columnar structures that grow almost perpendicular to the substrate and cover a significantly larger portion of the surface. At 1000 °C, the surface of the AlCrMoTaTi alloy is completely covered by a dense structure of thin whisker-like crystals, which does not resemble the morphology observed at lower temperatures.

In turn, Figure 12d presents the surface morphology of the AlCrNbTaTi alloy after sulfidation under conditions identical to those applied to the molybdenum-containing alloy at 1000 °C. The clearly visible similarities in the morphology of the scales formed under the same conditions on both investigated RHEAs suggest far-reaching similarities in their growth mechanisms. This would imply that either Mo or Nb influence on the sulfidation process of the discussed alloys is very similar.

**Figure 13:**
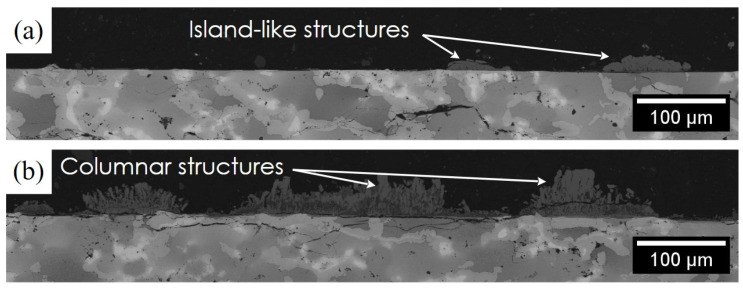
Microstructure of the scales formed on the AlCrMoTaTi alloy during isothermal sulfidation at (**a**) 800 °C and (**b**) 900 °C.

Figure 13 shows cross-sections of the Mo-containing alloy samples sulfidized at 800 and 900 °C. The microstructural differences clearly illustrate the localized nature of the structures protruding from the surface; outside these areas, the scale remains flat and very thin.

**Figure 14:**
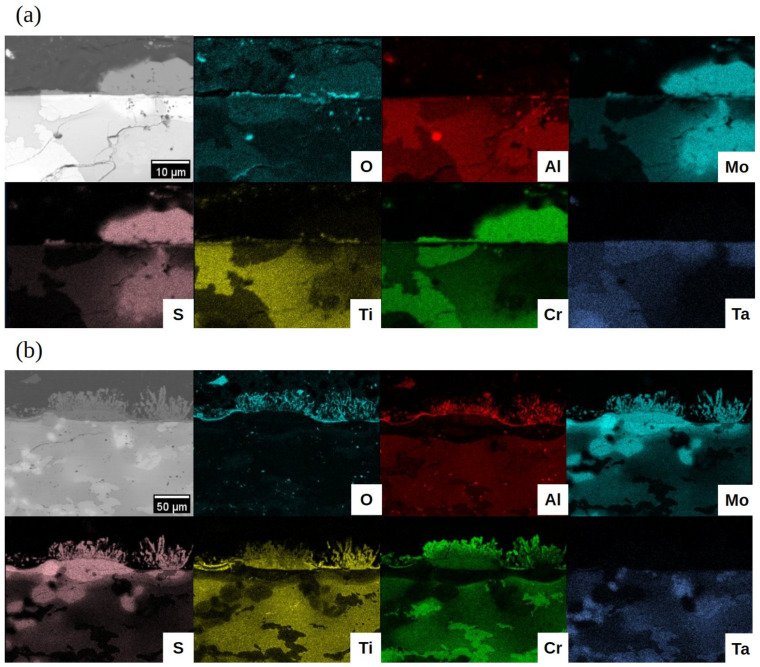
Elemental maps of the scales formed on the AlCrMoTaTi alloy isothermally sulfidized at (**a**) 800 °C and (**b**) 900 °C.

EDS elemental maps obtained from cross-sections of the AlCrMoTaTi alloy sulfidized at 800 and 900 °C—specifically in the regions featuring island-like and columnar structures, respectively (Figure 14a,b)—revealed that the former (grown at 800 °C) mainly consists of chromium sulfide. In contrast, the thin scale layer extending between these structures primarily contains Cr, Ti, and O. Meanwhile, the crystalline structures growing from the metallic substrate at 900 °C are composed of sulfur, Cr, Ti, and likely Mo.

Under the EDS measurement conditions, it was not possible to distinguish molybdenum from sulfur due to the overlap of signals (peak overlap) originating from these two elements. Therefore, all maps displaying molybdenum, especially within the areas occupied by reaction products, should be regarded only as a potential indication of this element’s presence in the analyzed region.

Interestingly, the surface of the columnar structures is covered with aluminum and oxygen, suggesting the presence of Al_2_O_3_ on the surface of the chromium and titanium sulfides. It is equally noteworthy that, based on the conducted analyses, no incorporation of tantalum from the substrate into the scale was detected.

**Figure 15:**
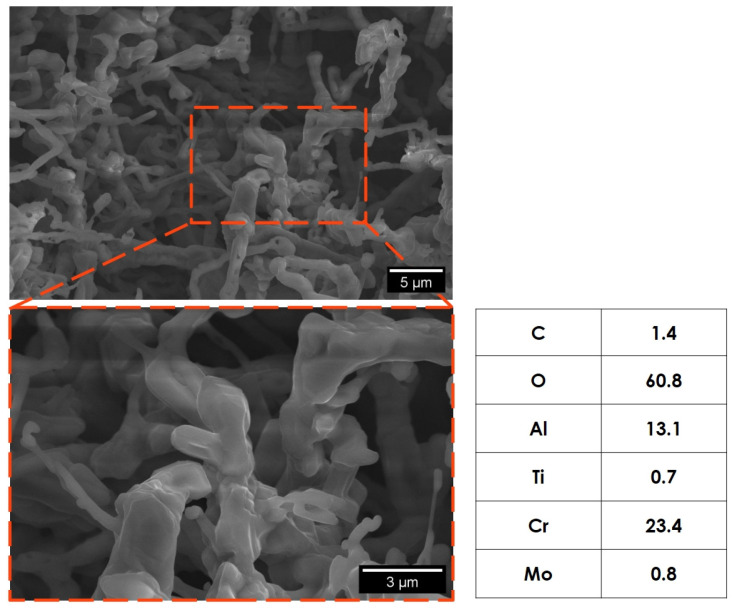
Whisker-like structure in the scale formed on the AlCrMoTaTi alloy sulfidized at 1000 °C for 100 h, and its average chemical composition (in at. %) determined by EDS inside the area indicated in the micrograph.

In turn, the whisker-like crystals forming the scale on the alloys sulfidized at 1000 °C are dominated by chromium, aluminum, and oxygen (Figure 15).

As previously mentioned, both aluminum and oxygen were present on the surface of the columnar structures that constituted the scale at 900 °C. This suggests that as process temperature increases, conditions become favorable for the formation of oxide-sulfide scale systems, with a greater fraction of oxide phases.

At this point, the presence of oxygen in scales formed in an atmosphere consisting of helium and sulfur requires clarification. Despite using ultra-high purity helium, oxygen is present in the system as a contaminant introduced by the carrier gas. Although the O_2_ content in 6.0 grade helium does not exceed 0.1 ppm, this amount is sufficient to make the oxidation of the investigated materials thermodynamically favorable under the reaction conditions described in this work.

## 4. Discussion

**Figure 16:**
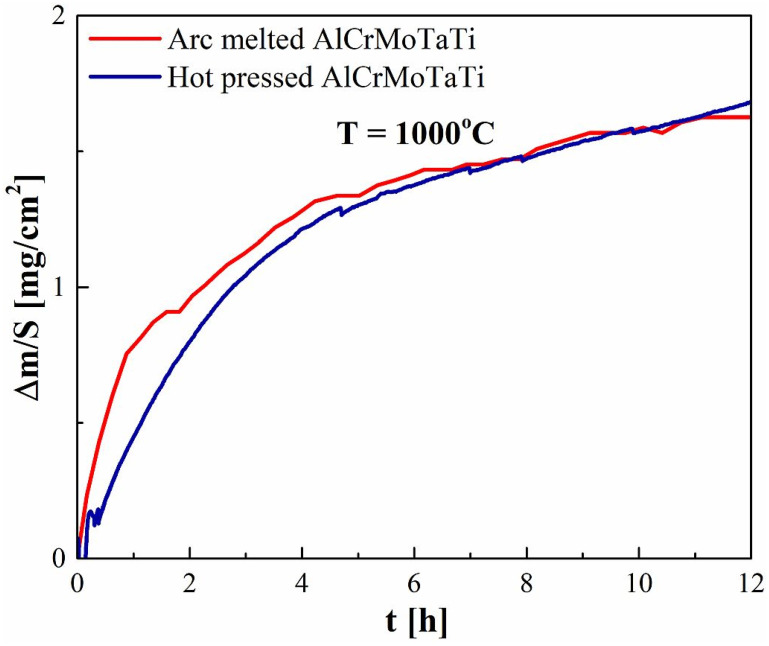
Kinetics curves obtained from arc-melted [12] and hot-pressed AlCrMoTaTi at 1000 °C in air atmosphere.

The SEM-EDS results obtained before the corrosion processes indicate non-uniformity of constituent element distribution, as significant separation of those elements can be observed in the alloys obtained via hot pressing, in contrast to counterparts synthesized via arc melting. From this it can be expected that the high-temperature corrosion products will be a multi-phase heterogeneous scale due to a lack of selective oxidation or sulfidation. Nevertheless, the mass gain received from thermogravimetric analysis of AlCrMoTaTi in this case is comparable with that achieved by Gorr et al. for arc-melted AlCrMoTaTi [12]. Figure 16 illustrates the respective kinetics curves obtained at 1000 °C for both hot-pressed and arc-melted AlCrMoTaTi up to 12 h. While the kinetics courses are similar, a key component in the oxide scale that Gorr et al. [12] claim is in large part responsible for the oxidation resistance of the arc-melted AlCrMoTaTi high-entropy alloy, i.e., CrTaO_4_, was not detected in the hot-pressed counterpart investigated in this work. Instead, XRD analysis seems to suggest that the presence of a previously undetermined Ta-Al spinel oxide phase is probable. It is possible that the similarity of the kinetic curves illustrated in Figure 16 results from the relatively short oxidation time (12 h), whereas the structural differences determined in Figure 9a may be due to the XRD studies being carried out after a longer oxidation period (100 h).

In any case, all the obtained results indicate that the oxidation properties of AlCrMoTaTi are vastly superior to those of AlCrNbTaTi. This can mainly be attributed to the growth of thicker Al_2_O_3_ and Cr_2_O_3_ scale layers on the AlCrMoTaTi alloy compared to the thin alternating layers observed in the oxide scales formed on AlCrNbTaTi, which did not provide the same level of corrosion protection. In fact, the lamellar scale formation resulted in linear oxidation after a certain duration, as opposed to parabolic. This occurrence has been previously determined during the oxidation of pure titanium, where, after a total weight gain of 2–5 mg/cm^2^, a parabolic to linear oxidation transition occurs [22]. This has been associated with the formation of lamellar rutile layers and attributed to large stresses and strains in the scale resulting in cracking and exfoliation of the metal’s outer layer, and subsequently, rapid oxidation [23]. Scale cracking and ‘breakaway’ linear oxidation have also been determined during high-temperature oxidation of niobium [24]. In this case, a lamellar Nb_2_O_5_ scale structure can also be seen [24]. Lamellar scale growth has also been identified in the case of Ti_3_Al binary alloy oxidized at 900 °C, in which case, below the outer TiO_2_ and Al_2_O_3_ layers, intermixed Al_2_O_3_ + TiO_2_ was observed [2]. Later studies demonstrated that a lamellar scale can also form during oxidation on more complex alloys, such as Ti_2_AlNb-based alloys [25]. Thus, it is reasonable to expect the same phenomenon in AlCrNbTaTi alloy as well.

A key factor to take into consideration when comparing the oxidation resistance of the two studied materials is the presence of Nb_2_O_5_ in the scale formed on the AlCrNbTaTi alloy. Studies have shown that niobium oxide is prone to cracking [24]. Subsequently, breakaway oxidation occurs and the kinetics transition from parabolic to linear [2], which explains the oxidation procedure of the AlCrNbTaTi samples. The poor protective properties of Nb_2_O_5_ can also be attributed to non-stoichiometric variants becoming thermodynamically favorable at elevated temperatures [26]. For example, studies have shown that at 950 °C oxygen deficit in Nb_2_O_5-x_ can be as large as x = 0.036 [27], resulting in oxygen vacancy pathways for inward oxygen diffusion through the scale to the substrate. Consequently, the inclusion of Nb_2_O_5_ in the protective scale leads to the inferior high-temperature corrosion resistance of the Nb-containing alloys compared to AlCrMoTaTi. The general negative impact of Nb addition and Nb_2_O_5_ formation has also been confirmed in other oxidized high-entropy alloy systems as well [28,29].

As for scale formation on the multi-component alloys in the oxidizing-sulfidizing atmosphere provided by He-S_2_ with O_2_ impurity, initially an oxide layer exclusively grows on the substrate surface. This is in accordance with previous studies on several traditional alumina- and chromia-forming alloys [7]. Subsequently, inward sulfur diffusion through the oxide scale occurs and isolated sulfide crystals begin to form at the alloy-scale interface. As the corrosion process progresses, these isolated sulfides grow, forming larger aggregates or even a continuous layer. Typically, this layer is located beneath the oxide scale. Generally, the better the high-temperature corrosion resistance of a given alloy, the later the complete penetration of the initially formed oxide scale occurs. Therefore, difficulties in identifying individual sulfide grains—or even the complete absence of a distinct sulfide layer within the reaction products—are not unusual. On the contrary, such a situation demonstrates the excellent high-temperature corrosion resistance of the alloy. It is worth noting that the presented progressive development of sulfides through the oxide scale is primarily related to the kinetics of the corrosion process, since from a thermodynamic standpoint under the applied experimental conditions, both oxides and sulfides of the individual constituent metals can form [13]. Furthermore, a scale morphology analogous to that described above also occurs during the corrosion of identical high-temperature corrosion-resistant high-entropy alloys produced via arc melting [13]. Attention should also be given to the role of refractory elements in improving the high-temperature sulfidation resistance of high-entropy alloys. This effect is evident when compared to a high-entropy alloy based on common metals [30] containing comparable amounts of Al and Cr, although it remains not fully defined at this stage of the investigation.

Scale morphology in oxidizing-sulfidizing conditions is temperature dependent. As temperature increases, the morphology of the resulting reaction products became less compact—evolving from dense island-like structures at 800 °C to whisker-like crystals covering the entire surface of the sample sulfidized at 1000 °C. Although the studied alloys exhibited a lack of both chemical and phase homogeneity, the fact that these specific morphological types were observed globally across the entire surface of a given sample demonstrates that the substrate inhomogeneities had a negligible impact on scale formation. In this situation, it appears that temperature was the sole governing factor determining the specific morphology by influencing the process kinetics. Based on these observations, a valuable additional conclusion can be drawn regarding the scale formation mechanism via outward diffusion of substrate components to the surface; only such a mechanism can account for the growth of structures that are highly extended in the direction normal to the metallic core surface. This exact mechanism has been proposed in studies on the formation of whiskers, including alumina (Al_2_O_3_), on alumina-forming alloys [31,32].

**Figure 17:**
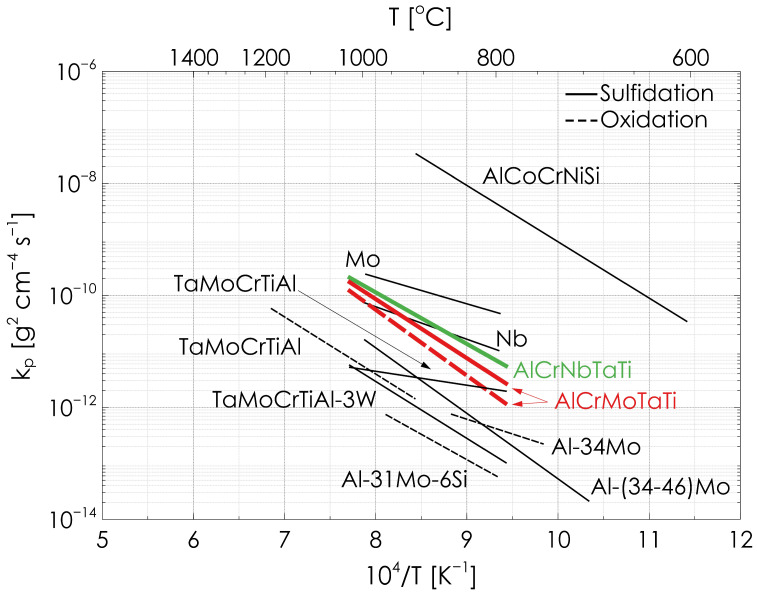
Sulfidation resistance of AlCrMoTaTi and AlCrNbTaTi alloys compared to literature data for high-temperature sulfur-corrosion-resistant materials and a high-entropy alloy containing no refractory elements. Literature data for comparison are taken from references [12] (TaMoCrTiAl), [13] (TaMoCrTiAl and TaMoCrTiAl-3W), and [30] (AlCoCrNiSi); the remaining datasets are based on [33].

A comparison of our results with literature data (Figure 17) for Al–Mo-type alloys (as well as pure molybdenum and niobium—elements known for their high resistance to high-temperature sulfidation) indicates that the refractory multi-component alloys fabricated by hot pressing exhibit significant sulfidation resistance in the temperature range below 1000 °C. This resistance clearly exceeds that of the pure metals and is only slightly inferior to the currently best-known sulfidation-resistant Al–Mo alloys and the TaMoCrTiAl alloy produced by arc melting. Crucially, the Mo-containing alloy described in this work, produced via powder sintering, exhibits practically identical resistance to both high-temperature sulfidation and oxidation. This confirms the hypothesis regarding the potential to develop materials resistant to aggressive atmospheres with both oxidizing and sulfidizing properties.

It is worth noting that the multi-component alloys described in this study, which contain refractory metals, aluminum, and chromium, significantly outperform the AlCrCoNiSi alloy in terms of sulfidation resistance [30]. This demonstrates that substituting common conventional metals, such as cobalt or nickel, with refractory metals in the high-entropy alloy composition is crucial for enhancing high-temperature sulfidation resistance.

## 5. Conclusions

Hot pressing, as a fabrication method for refractory multi-component/high-entropy alloys, represents a viable alternative to the conventional arc melting of constituent elements. The materials produced by this route exhibit good oxidation resistance and satisfactory resistance to high-temperature sulfidation. This behavior is attributed to the formation of a protective scale composed of Al_2_O_3_ and Cr_2_O_3_ under oxidizing conditions, and a complex oxide–sulfide scale in a sulfidizing atmosphere, as clearly demonstrated by the results presented in this study. Both oxidation and sulfidation of the AlCrMoTaTi and AlCrNbTaTi alloys generally follow a parabolic rate law, indicating diffusion-controlled kinetics through the scale. Under both oxidizing and sulfidizing conditions, the Mo-containing alloy exhibits superior high-temperature corrosion resistance.

The successful development of oxidation- and sulfidation-resistant multi-component alloys via a powder metallurgy-based approach demonstrates the potential for employing other powder-based techniques, such as thermal spraying, to produce protective coatings from the investigated materials.

## Figures and Tables

**Table 1:** Chemical compositions identified from EDS point analysis at the surface of AlCrMoTaTi and AlCrNbTaTi multi-component alloys after oxidation at 900 °C.

Sample	Point	Element [at%]						
		Al	Cr	Ta	Ti	Mo	Nb	O
AlCrMoTaTi	1	5.1	3.7	0.4	29.3	1.3	0	60.2
	2	9.5	1.7	16.6	2.0	0	0	70.3
	3	29.4	1.6	0.2	1.3	0	0	67.5
	4	17.4	12.3	2.3	14.7	0	0	53.3
	5	12.6	26.3	3.8	4.2	0	0	53.2
AlCrNbTaTi	1	10.0	5.3	2.4	14.4	0	5.4	62.4
	2	8.0	2.7	12.6	2.0	0	1.0	73.7
	3	1.3	0.1	0.3	20.6	0	0.3	77.3
	4	3.2	5.9	0.3	0.9	0	10.7	78.9
	5	7.7	6.0	0.8	2.7	0	14.6	68.1
	6	7.1	28.9	3.8	7.8	0	2.9	49.5

## Data Availability

The original contributions presented in this study are included in the article. Further inquiries can be directed to the corresponding author.

## Appendix A

**Table A1:** Parabolic oxidation rate constant and corresponding fitting parameters of the linear regression used to determine k_p_ values.

Material	Temperature [°C]	k_p_ [g^2^ cm^−4^ s^−1^]	Fitting Range [h]	R^2^ [-]
AlCrMoTaTi	800	1.3 × 10^−12^	30–100	0.743 *
	900	2.0 × 10^−11^	30–100	0.997
	1000	6.6 × 10^−11^	10–100	0.998
AlCrNbTaTi	800	2.8 × 10^−11^	2–35	0.996
	900	-	-	-
	1000	4.6 × 10^−10^	30–100	0.998

* R^2^ is inadequate here because when the effect of X on Y is extremely small, R^2^ reflects residual noise rather than model fit.

**Table A2:** Parabolic sulfidation rate constant and corresponding fitting parameters of the linear regression used to determine k_p_ values.

Material	Temperature [°C]	k_p_ [g^2^ cm^−4^ s^−1^]	Fitting Range [h]	R^2^ [-]
AlCrMoTaTi	800	2.9 × 10^−12^	25–100	0.994
	900	3.6 × 10^−11^	30–90	0.998
	1000	9.8 × 10^−11^	40–100	0.999
AlCrNbTaTi	800	7.5 × 10^−12^	0–98	0.999
	900	3.1 × 10^−11^	25–100	0.998
	1000	1.7 × 10^−10^	30–100	0.993

## Appendix B

**Table A3:** PDF card information on the phases identified from patterns obtained before oxidation.

Identified Phase	PDF Number
Mo_0.67_Ti_0.33_	96-152-3263
Al_3.1_Mo_0.9_	96-152-2872
AlTi	96-153-2770
Cr_0.5_Mo_0.5_	96-152-4009
Nb_0.3_Ti_0.7_	96-152-2551
Cr_2_Ti	96-152-5361
Al_69_Ta_39_	96-152-7750
NbTa	96-152-3087

**Table A4:** PDF card information on the phases identified from patterns obtained after oxidation.

Identified Phase	PDF Number
Al_2_O_3_ (corundum)	96-101-0952
Ta_2_O_5_	96-154-0127
AlTaO_4_	96-200-2308
TiO_2_ (rutile)	96-900-9084
Cr_2_O_3_	96-901-4811
Nb_2_O_5_	96-153-4157

**Table A5:** Miller indices of peaks corresponding to the Mo_0.67_Ti_0.33_ phase identified in the XRD pattern obtained from AlCrMoTaTi before oxidation.

*2θ* [°]	*h*	*k*	*l*
40.17	1	0	1
58.11	2	0	0
72.99	2	1	1
86.75	2	0	2
100.32	3	0	1

**Table A6:** Miller indices of peaks corresponding to the Al_3.1_Mo_0.9_ phase identified in the XRD pattern obtained from AlCrMoTaTi before oxidation.

*2θ* [°]	*h*	*k*	*l*
25.47	1	0	1
36.34	2	0	0
40.80	2	0	1
44.90	2	1	1
52.33	2	0	2
68.40	2	3	0
71.37	3	2	1
77.16	4	0	0
91.19	4	2	1

**Table A7:** Miller indices of peaks corresponding to the AlTi phase identified in the XRD pattern obtained from AlCrMoTaTi before oxidation.

*2θ* [°]	*h*	*k*	*l*
21.90	0	0	1
38.72	1	0	1
44.65	0	0	2
65.41	1	1	2
69.47	0	0	3
113.64	3	0	1

**Table A8:** Miller indices of peaks corresponding to the Cr_0.5_Mo_0.5_ phase identified in the XRD pattern obtained from AlCrMoTaTi before oxidation.

*2θ* [°]	*h*	*k*	*l*
42.58	1	0	1
61.79	2	0	0

**Table A9:** Miller indices of peaks corresponding to the Nb_0.3_Ti_0.7_ phase identified in the XRD pattern obtained from AlCrNbTaTi before oxidation.

*2θ* [°]	*h*	*k*	*l*
36.83	0	2	0
38.30	0	0	2
39.41	1	1	1
41.71	0	2	1
59.65	2	0	0
71.58	0	2	3
93.28	1	3	3
102.53	3	1	1
110.32	2	4	1
115.17	1	5	1

**Table A10:** Miller indices of peaks corresponding to the Cr_2_Ti phase identified in the XRD pattern obtained from AlCrNbTaTi before oxidation.

*2θ* [°]	*h*	*k*	*l*
21.53	1	0	1
30.61	0	1	4
35.02	0	1	5
42.71	2	0	1
43.09	1	1	4
45.76	0	2	3
59.19	0	2	7
74.00	0	2	10
79.73	0	2	11
82.28	1	3	2
83.61	1	3	3
85.90	0	2	12
102.09	0	4	6
111.41	1	2	14
117.72	2	3	7

**Table A11:** Miller indices of peaks corresponding to the NbTa phase identified in the XRD pattern obtained from AlCrNbTaTi before oxidation.

*2θ* [°]	*h*	*k*	*l*
38.58	1	0	1
69.81	2	1	1

**Table A12:** Miller indices of peaks corresponding to the Al_69_Ta_39_ phase identified in the XRD pattern obtained from AlCrNbTaTi before oxidation.

*2θ* [°]	*h*	*k*	*l*
20.74	4	0	2
22.75	2	4	2
26.32	4	0	4
32.38	4	4	4
33.41	7	1	1
36.02	7	1	3
40.80	5	7	1
54.86	11	1	3
56.66	11	3	3
62.05	8	10	0
66.19	12	2	6
66.79	13	3	3
76.39	14	2	6

**Table A13:** Miller indices of peaks corresponding to the TiO_2_ phase identified in the XRD patterns obtained from AlCrMoTaTi and AlCrNbTaTi after oxidation.

*2θ* [°]	*h*	*k*	*l*
27.46	1	1	0
36.12	1	0	1
41.27	1	1	1
54.37	2	1	1
56.68	2	2	0
62.83	0	0	2
64.11	1	3	0
69.07	3	0	1

**Table A14:** Miller indices of peaks corresponding to the AlTaO_4_ phase identified in the XRD patterns obtained from AlCrMoTaTi and AlCrNbTaTi after oxidation.

*2θ* [°]	*h*	*k*	*l*
27.38	1	1	0
35.84	1	0	1
39.11	2	0	0
41.01	1	1	1
54.09	2	1	1
56.51	2	2	0
63.91	1	3	0
68.75	3	0	1
75.98	2	0	2
82.05	3	2	1

**Table A15:** Miller indices of peaks corresponding to the Ta_2_O_5_ phase identified in the XRD patterns obtained from AlCrMoTaTi and AlCrNbTaTi after oxidation.

*2θ* [°]	*h*	*k*	*l*
22.83	0	1	0
28.27	12	0	1
28.76	0	0	2
37.03	0	1	2
44.20	3	0	3
50.64	12	0	3
55.45	12	2	1

**Table A16:** Miller indices of peaks corresponding to the Cr_2_O_3_ phase identified in the XRD patterns obtained from AlCrMoTaTi and AlCrNbTaTi after oxidation.

*2θ* [°]	*h*	*k*	*l*
24.84	1	0	2
50.95	2	0	4
59.29	2	1	2
74.04	1	0	10

**Table A17:** Miller indices of peaks corresponding to the Al_2_O_3_ phase identified in the XRD patterns obtained from AlCrMoTaTi and AlCrNbTaTi after oxidation.

*2θ* [°]	*h*	*k*	*l*
25.60	1	1	0
35.17	2	1	1
43.39	2	1	0
61.34	3	3	2
95.34	4	2	0
101.16	5	3	2

**Table A18:** Miller indices of peaks corresponding to the Nb_2_O_5_ phase identified in the XRD patterns obtained from AlCrMoTaTi and AlCrNbTaTi after oxidation.

*2θ* [°]	*h*	*k*	*l*
29.99	3	−1	1
33.37	0	2	0
50.55	0	2	2
